# Identification and Assessment of Potential Drug-Drug Interactions in Intensive Care Unit Patients

**DOI:** 10.5005/jp-journals-10071-23147

**Published:** 2019-04

**Authors:** Bhavika Ravindra Wagh, Deepa Dhananjay Godbole, Shubham Shivaji Deshmukh, Shivakumar Iyer, Prasanna R Deshpande

**Affiliations:** 1-3,5 Department of Clinical Pharmacy, Poona College of Pharmacy, Bharati Vidyapeeth Deemed University, Pune, Maharashtra, India; 4 Department of Critical Care Medicine, Bharati Hospital and Research Centre, Pune, Maharashtra, India

**Keywords:** Intensive care unit, Monitoring and assessment, Potential drug-drug interaction

## Abstract

**Context:**

Intensive care unit (ICU) patients suffer from various comorbidities and usually receive complex pharmacotherapy which increases the risk of drug-drug interactions (DDIs).

**Aim:**

To identify and assess potential DDIs (pDDIs) in ICU patients.

**Settings and design:**

A prospective observational study conducted in ICU of a tertiary care hospital for a period of 6 months.

**Materials and methods:**

Patient information was noted in the data collection form and pDDIs were assessed using Micromedex®database.

**Statistical analysis used:**

Chi-square test was used to find correlation of pDDIs with patient parameters. *p* value was calculated keeping the significance level 0.05.

**Results:**

Total 400 subjects were included; having an average age of 55.99 ± 15.62 years with a higher percentage of males (61.75%). About 305 (76.25%) patients were found with pDDIs, showing an average of 2.93 pDDIs/patient. The findings of this study were as follows: Total interactions = 1171, contraindicated = 6 (1%), major = 715 (61%), moderate = 428 (36%), and minor = 22 (2%) pDDIs. Further, majority of pDDIs had onset of action “not specified” documentation “fair” and probable mechanism “pharmacodynamic” in nature. Significant association of occurrence of pDDIs was found with number of drugs prescribed to patients in ICU.

**Conclusion:**

This study demonstrated a high prevalence of pDDI in ICU due to the complexity of pharmacotherapy which showed major pDDIs as the most evident (61%) while contraindicated were 1%. Further studies are needed to better explore this area which may help in realizing the goal of good clinical practice and may offer a methodology to further increase drug safety.

**Key messages:**

“Monitoring and assessment of DDIs is needed for better patient care”.

**How to cite this article:**

Wagh BR, Godbole DD, *et al.* Identification and Assessment of Potential Drug-Drug Interactions in Intensive Care Unit Patients. Indian J Crit Care Med 2019;23(4):170-174.

## INTRODUCTION

Drug-drug interaction is a pharmacological or clinical response to the administration of two or more drugs, which is different from the response triggered by the individual use of these agents.^1^ This interaction can cause reduced, null or increased drug effect. When the interactions present in the prescription are theoretically evaluated through databases and not by their actual occurrence, they are considered pDDIs.^1^ The risk factors that contribute to the occurrence of pDDIs include patients receiving intensive care, immunosuppressed patients, patients with complex clinical condition which need large number of prescription drugs with long duration of hospital stay, and increase in healthcare costs.^2^ The abovementioned risk factors are associated with patients in ICU settings thus they are at greater risk for experiencing pDDIs.^3^ A study done in ICU showed that risk of a pDDI increases by approximately 6% per day.^3^ Often pDDIs go unnoticed in these patients as their symptoms due to disease mask the symptoms caused by pDDIs. Within the context of above facts, it is important to investigate pDDIs as there are few studies available and to spread the awareness for the same, thereby improving patient safety in ICU settings of Indian hospitals. Hence, we aimed to determine the prevalence of pDDIs in the ICU setting.

## SUBJECTS AND METHODS

A prospective observational study was done in tertiary care teaching hospital. This study was conducted for a period of 6 months from September 2017 to February 2018. Institutional Ethics Committee approval was taken before commencing the study.

Case records of all the patients above 18 years of age, length of stay up to the day of discharge, and prescriptions having more than two medications prescribed were included in the study. Medicolegal cases were excluded. Data were collected on the data collection form which included details about patient's demographic, provisional diagnosis, prescription details, and number of days in hospital. The data were analyzed for pDDIs by using drug interaction software Micromedex® database.

**Table 1:** Severity scale^4^

*Severity*	*Description*
Contraindicated	The drugs are contraindicated for concurrent use
Major	The interaction may be life-threatening and/or require medical intervention to minimize or prevent serious adverse effects.
Moderate	The interaction may result in exacerbation of the patient's condition and/or require an alteration in therapy.
Minor	The interaction would have limited clinical effects. Manifestations may include an increase in the frequency or severity of the side effects but generally would not require a major alteration in therapy.

**Fig. 1:**
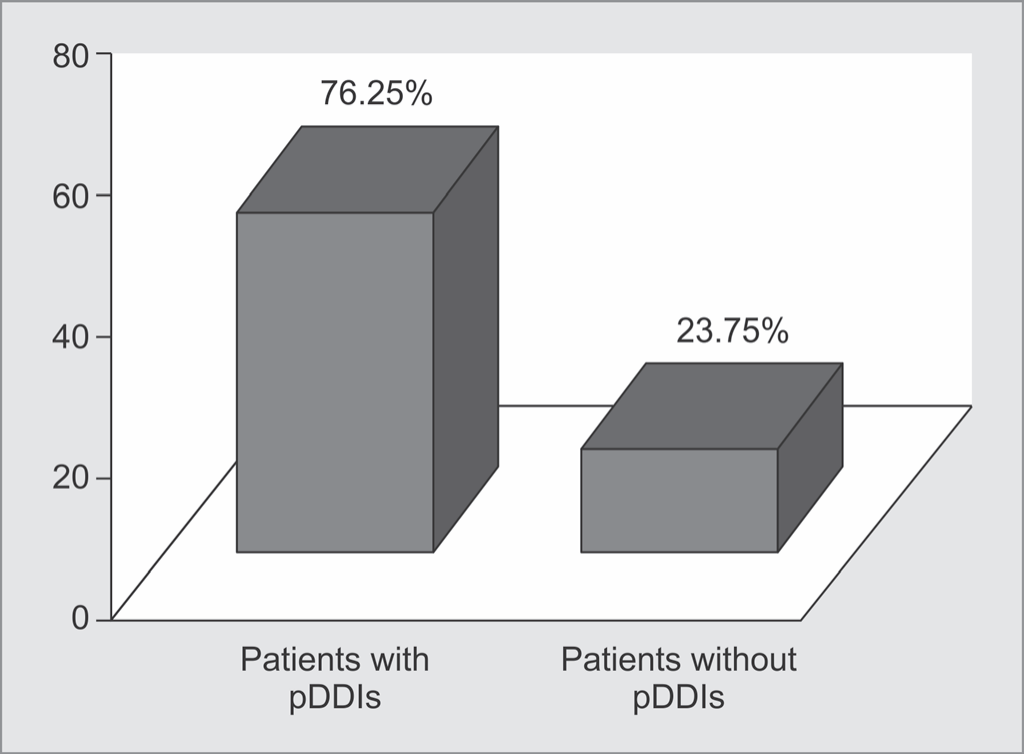
Distribution of pDDIs in ICU patients

**Fig. 2:**
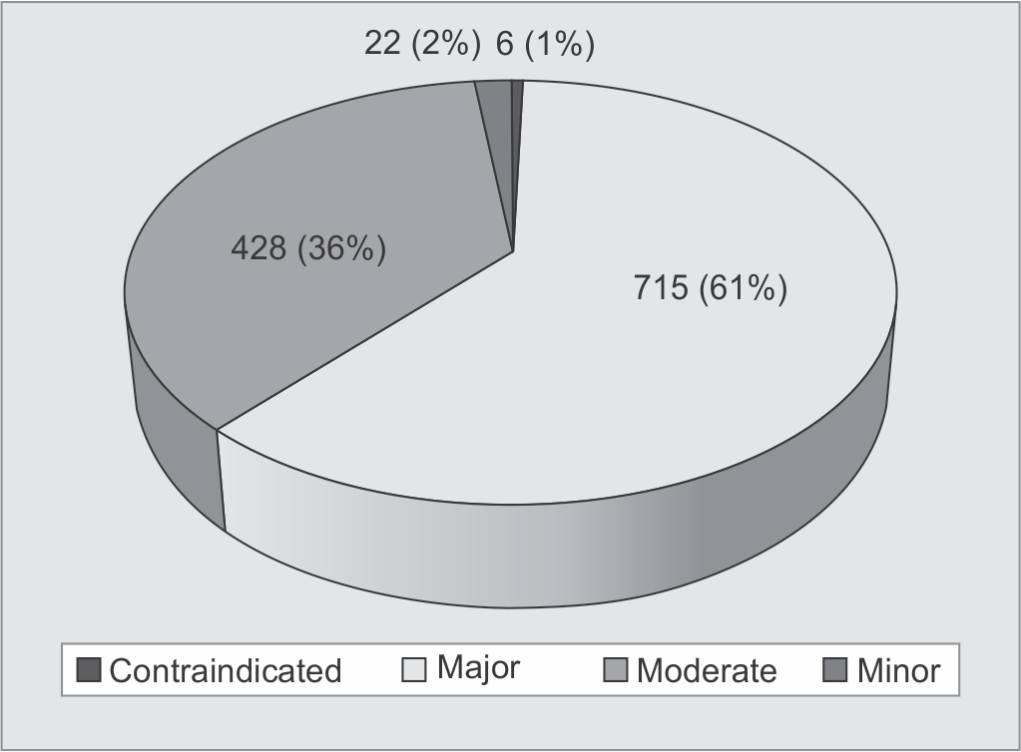
Classification of interaction by severity

Micromedex® database classifies pDDIs according to the severity scale (Table 1), mechanism [pharmacokinetic (PK) and pharmacodynamics (PD)], onset of action (rapid, delayed, not specified), and documentation.^4^

For statistical analysis of data, Chi-square calculator^5^ was used for determining the statistical significance between pDDIs and patient parameters like age, gender, length of stay, and number of drugs. Association of the variables with pDDIs was checked by calculating the *p* value keeping the significance level <0.05.

## RESULTS

Data from 400 ICU patients were collected. Raosoft calculator was used to calculate margin of error. Parameters considered were confidence level (95%) and response distribution (50%). Margin of error with a sample size of 400 was found to be 4.85%.^6^

Out of 400 patients included in the study, 247 (61.75%) were males and 153 (38.25%) were females. The average age of patients in years was 55.99 ± 15.62. The average length of stay of patients in hospital was 5.65 ± 5.42. The average number of drugs per prescription was 8.8 ± 3.35.

Total number of generic drugs prescribed in ICU patients was 3,520. As far as route of administration (ROA) was considered, the patients received medicines mostly via the intravenous (46%) and oral (45%) routes. Other ROA were subcutaneous, respiratory therapy, infusion, and nebulization.

Regarding frequency of the administered drugs, majority of them were once daily, twice daily, and thrice daily. A total of 1,171 interactions were found showing an average of 2.93 pDDIs/patient. Distribution of pDDIs in ICU patients is shown in Figure 1.

Average pDDI per 400 patients was found to be: contraindicated 0.02, major 1.79, moderate 1.07, and minor 0.06. Formula: Average pDDI = Number of interactions (each severity)/400 patients. Each severity distribution of pDDIs seen in ICU patients is shown in Figure 2.

Assessment of most frequently seen interactions was done. Most frequently interacting individual drugs in each severity category are shown in Table 2. Most commonly seen pDDIs are shown in Table 3. On review of adverse effects of pDDIs as per system-wise distribution, cardiological (n = 326) and hematological (n = 313) were found to be most common and evident. Occurrence of pDDIs as per the distribution of adverse effects are shown in Table 4.

In regard to the assessment of pDDIs in this study, the majority of onset of action for pDDIs was found to be “not specified” (70.09%). The documentation for major pDDIs in patients was found to be fair (61.54%). Further majority of the probable mechanism for pDDIs was pharmacodynamic (73.89%) in nature. The major clinical management for pDDIs identified was monitoring signs and symptoms and monitoring laboratory parameters (Table 5).

References for pDDIs were calculated as per Micromedex® database (Table 6). Further, Chi-square statistical calculator was used to calculate *p* value and determine the significant statistical association. Significant statistical association of pDDIs was found with number of drugs prescribed to patients in ICU. Data were grouped for each parameter as following: Age (18-25, 26-50, 51-75, 76-100), length of stay (1-15, 16-30, 31-45, 46-60, 61-75), and number of drugs (1-15, 16-30) (Table 7).

## DISCUSSION

To the best of our knowledge, this is one of the rare studies that were conducted in India for identification and assessment of pDDIs in ICU patients.^3, 7, 8^

**Table 2:** Individual drugs frequently interacted

*Severity*	*Number of drugs (n)*	*%*
*Contraindicated*		
Fluconazole	3	25
Ondansetron	3	25
Linezolid	2	16.67
*Major*		
Aspirin	214	0.15
Ondansetron	205	0.14
Clopidogrel	162	0.11
Azithromycin	78	0.05
Metronidazole	54	0.04
Heparin	55	0.04
Furosemide	44	0.03
*Moderate*		
Atorvastatin	94	0.11
Aspirin	80	0.09
Clopidogrel	79	0.09
Furosemide	44	0.05
Phenytoin	44	0.05
Metoprolol	36	0.04
Pantoprazole	35	0.04
*Minor*		
Aspirin	6	0.14
Furosemide	4	0.09
Phenytoin	4	0.09
Hydrocortisone	3	0.07

**Table 3:** Most commonly seen pDDIs

*pDDI*	*Number of drugs (n)*	*%*
*Contraindicated*		
Fluconazole + Ondansetron	3	50
*Major*		
Aspirin + Clopidogrel Ondansetron + Azithromycin Ondansetron + Metronidazole Aspirin + Furosemide Heparin + Aspirin	70 59 45 40 21	9.79 8.25 6.29 5.59 2.94
*Moderate*		
Atorvastatin + Clopidogrel Atorvastatin + Azithromycin Aspirin + Metoprolol Aspirin + InsulinHR Digoxin + Furosemide	65 22 17 15 14	15.19 5.14 3.97 3.50 3.27
*Minor*		
Aspirin + Hydrocortisone Furosemide + Phenytoin Azithromycin + Magnesium hydroxide	3 2 2	13.64 9.09 9.09

As for the prevalence of pDDIs in ICU settings, some studies found prevalence in the range of 70-90%.^1–3,7–11^ Similarly, in our study, prevalence of pDDIs was found to be 76.25%. Thus, the need for their evaluation and monitoring is evident.

Various databases were used in previous studies like Micromedex®, ^1,2,11–14^ DrugReax software,^10^ Medscape drug interaction checker,^3,14^ Lexi comp,^3,7,8,14^ drug interactions fact, 2010 textbook.^15^ We used the Micromedex® database.

Considering the sociodemographic characteristics, many studies had male preponderance which is consistent with the present study.^1,3,7,8,11,14,15^

Drug-drug interactions are more frequent in patients over 60 years of age because they suffer from chronic conditions requiring multidrug therapy. The elderly are also more susceptible to DDIs due to presence of liver and kidney dysfunction, as well as reduced drug metabolism and elimination.^2^ Thus, elderly patients are at a greater risk for developing pDDIs. Present study found 167 patients above 60 years of age with 518 (44.24%) pDDIs, these findings were in concordance with previous studies.^2, 3, 10^

As far as hospital stay is considered, in few studies, average hospital stay was found to be 5-6 days similar to present study. ^7, 8, 10^

In regard to the number of drugs prescribed, average number of drugs prescribed in Rodrigues et al.^1,11^ was 13, Hamidy and Fauzia^7^ was 7, Gupta et al.^3^ was 8.25, Rafiei et al.^15^ was 5.6 and in Abideen et al.^8^ were 17.09. Average number of drugs prescribed in this study was found to be 8.8 drugs per prescription.

In regard to the most common drugs prescribed, studies revealed use of ranitidine,^2,15^ cefepime,^2^ furosemide,^2^ fentanyl,^2^ and phenytoin.^15^ Unlike these studies, this study showed ondansetron (n = 334), pantoprazole (n = 322), and aspirin (n = 139) as most prescribed drugs.

With respect to the route of drug administration, some of the studies have similarly pointed to intravenous injection as the most frequent way of administration of drugs, same as our study.^2,15^ It is the preferred route in ICU patients, since most patients require a fast route for immediate drug effects.^2^ In present study, a large majority of the patients (46%) received medications via the intravenous route and least preferred route was respiratory therapy (0.77%).

Severity is one of the major aspects to be considered while monitoring the pDDIs. Previous studies reported moderate pDDIs in the range of 65-75%.^1–3,7,8,11,15,16^ Unlike these studies, we found a high number of major pDDIs (61%).

Most frequently interacting individual drugs in previous studies were found to be phenytoin,^7, 8, 15^ dexamethasone,^9^ midazolam,^2,10^ and furosemide.^7^ We found that the top two most frequently interacting individual drugs in each severity category to be as follows:

Contraindicated: Fluconazole (25%), ondansetron (25%), Major: Aspirin (0.15%), ondansetron (0.14%), Moderate: Atorvastatin (0.11%), aspirin (0.09%) and Minor: Aspirin (0.14%) and phenytoin (0.09%).

We found that nearly half of the drugs with pDDIs to be cardiovascular drugs as a large proportion of the patients admitted to the ICU were diagnosed with cardiovascular diseases (n = 136).

To the best of our knowledge, Rodrigues et al.^1,11^ was the only study which quantified contraindicated pDDIs (n = 12). The most commonly seen contraindicated interactions in their study were in the presence of metoclopramide (79.4%). Our study identified contraindicated pDDIs with fluconazole and ondansetron most often (50%).

Major and moderate pDDIs were the most frequently reported pDDIs by previous studies. Major pDDIs identified in previous studies were: enoxaparin + dipyrone (n = 132),^2,11^ midazolam + fentanyl (n = 103),^2,10^ ranitidine + phenytoin (n = 8),^15^ phenytoin + dopamine (n = 7),^15^ clopidogrel + pantoprazole (n = 19),^7^ hydrocortisone + ofloxacin (n = 6),^8^ aspirin + beta blocker (n = 30).^3^

**Table 4:** Distribution of pDDIs as per adverse effects

*Clinical system*	*Contraindicated (n)*	*Major (n)*	*Moderate (n)*	*Minor (n)*
Cardiologic	5	219	103	0
Hematologic	0	261	52	0
Toxicity	0	106	58	1
Renal	0	62	1	0
Reduced drug effectiveness	0	28	97	7
Hepatic	0	11	10	0
Neurologic	0	12	0	0
Electrolyte imbalance	0	6	20	0
Metabolic/endocrine	0	5	32	0
Musculoskeletal	0	5	20	0
Respiratory	0	4	0	0
Others^*^	1	0	38	12

^*^reduced iron bioavailability (n=9), increased INR or prothrombin time (n=11), increased GI ulceration (n=10), postural hypotension (n=12), alteration in drugs own action (n=6), increased plasma concentration of CYP2C19 substrate (n=3).

**Table 5:** Management parameters for pDDIs*

*Management parameters*	*Contraindicated*	*Major*	*Moderate*	*Minor*
Monitoring signs and symptoms	0	288	128	6
Monitoring laboratory parameters	4	372	169	5
Change in dose	1	91	78	5
Change in drug	2	129	114	5
Avoid concurrent administration of interacting drugs	0	31	0	0
Change in time	0	22	18	4

* The total count is fluctuated as the management parameters were found to be more than one for some of pDDIs.

**Table 6:** References of pDDIs as per Micromedex®

*Reference*	*Count*	*Average per 400 patients*
Contraindicated	13	0.03
Major	2079	5.2
Moderate	1623	4.06
Minor	58	0.15

Unlike these studies we found different drug combinations with major pDDIs such as aspirin + clopidogrel (n = 70) followed by ondansetron + azithromycin (n = 58).

Commonly seen moderate pDDIs as per other studies were: furosemide + hydrocortisone (6.9%),^10^ insulin + acetylsalicylic acid (17.3%),^1,11^ dopamine + noradrenaline (6.6%).^8^ Whereas, in the same manner as our study, Siddiqui et al.^7^ also reported frequent occurrence of moderate pDDI: atorvastatin + clopidogrel. Other moderate pDDIs found in present study were atorvastatin + azithromycin (5.14%) followed by aspirin + metoprolol (3.97%).

Minor pDDIs were rarely reported in previous studies.^3,7^ However, present study reported commonly seen minor pDDIs: aspirin + hydrocortisone (13.64%) followed by furosemide + phenytoin (9.09%).

Onset of action is also an important parameter in assessment of pDDIs. Most pDDIs identified in study by Lima and Cassiani^2^ had slow onset (55.4). Mechanism of action for majority of pDDIs were PK (48.2%) followed by PD interactions (44.4%) and 7.4% were classified as unknown, i.e. the underlying mechanism of interaction was not clear. We found in present study that regardless of severity, majority of the pDDIs had non-specified onset of action (70.08%), fair documentation (61.54%). Majority of pDDIs were PD (73.89%) followed by PK interactions (20.64%) and 5.46% were unknown mechanisms.

To avoid and treat the pDDIs, previous investigations recommended the following:

- Avoidance of drug combination or concomitant use^1,2,8,14,10^
- Monitoring signs and symptoms^2^
- Dose adjustments^1, 2, 10^
- Therapy modification/replacement of drug.^2, 8, 14^

In addition, we recommend monitoring laboratory parameters and change in time of administration of one of the interacting drugs.

References allow you to acknowledge the depth to which the information was collected or research was conducted. References acknowledge the source of information and assure its evidence, reliability, and specificity. Therefore, evaluation of references is an important aspect which was considered while assessing pDDIs.

References for pDDIs as per Micromedex®: Contraindicated pDDIs had n = 13, major pDDIs n = 2079, moderate pDDIs n = 1623, and minor pDDIs had n = 58. To the best of our knowledge, this is the only Indian study, which has evaluated references for each severity category of pDDIs.

**Table 7:** Statistical data

*Parameters*	*Categories*	*Total (n)*				*p value*	*Chi-square*	*Degree of freedom*
		*Contra*	*Major*	*Moderate*	*Minor*			
Age	18-25 26-50 51-75 76-100	0 1 5 0	19 112 211 33	4 54 124 12	0 6 9 4	0.1994	12.2541	9
Gender	M F	3 3	232 143	125 69	9 10	0.4605	2.5829	3
Length of stay	1-15 16-30 31-45 46-60 61-75	5 1 0 0 0	362 10 0 1 1	185 8 1 0 1	18 1 0 0 0	0.8367	7.3072	12
Number of drugs	1-15 16-30	5 1	361 14	184 10	16 3	0.04634	7.98414	3

With respect to the statistical correlation, various studies had shown a direct statistical relationship between pDDIs and increase in number of drugs prescribed.^1–3,10,14,15^ The present study also showed a significant statistical association between number of drugs prescribed and number of pDDIs (*p* = <0.05). However, this study displayed no significant association between the number of pDDIs with any other parameters.

## CONCLUSION

This study highlights high prevalence of pDDIs in ICU settings. Major pDDIs were high in proportion in our study. The role of clinical pharmacist is crucial to identify and assess the pDDIs in ICU settings. Further studies are needed to better explore this area which may help in realizing the goal of good clinical practice and may offer a methodology to further increase drug safety.
